# SARS-CoV-2 testing for asymptomatic adult cancer patients before initiating systemic treatments: a systematic review

**DOI:** 10.3332/ecancer.2020.1100

**Published:** 2020-09-09

**Authors:** Guilherme Haradaa, Fernanda F Antonacio, Aline BL Gongora, Marina H Behar, Fernanda C Capareli, Diogo A Bastos, Rodrigo R Munhoz, Frederico P Costa, Denis L Jardim, Celso Arrais-Rodrigues, Yana Novis, Artur Katz, Gilberto de Castro Junior

**Affiliations:** Oncology Centre, Hospital Sírio-Libanês, Rua Dona Adma Jafet, 91, São Paulo 01308-050, Brazil; a https://orcid.org/0000-0003-4012-3251

**Keywords:** severe acute respiratory syndrome-related coronavirus, COVID-19 diagnostic testing, neoplasms, treatment

## Abstract

**Introduction:**

Cancer patients may have a higher risk of severe events and unfavourable outcomes in the setting of COVID-19. This review addresses the question of whether to test asymptomatic cancer patients before initiating systemic cancer treatments.

**Methods:**

This systematic review was conducted based on the PRISMA framework. Pubmed, Embase, Web of Science and Cochrane Central Register of Controlled Trials were systematically searched, as well as guidelines from international institutions involved in cancer care and COVID-19 research. Studies published in English, from 1 December 2019 to 27 May 2020 were considered eligible. We included studies which mentioned testing strategies for SARS-CoV-2 of asymptomatic cancer patients before starting immunosuppressive treatments.

**Results:**

We identified 1,163 studies and 4 guidelines through the literature search. A total of 18 articles were considered eligible and were included in the final analysis. Two articles were cohort studies, and the remaining were expert consensuses and published guidelines. The most common recommendation among the studies in this systematic review was to test asymptomatic patients for SARS-CoV-2 prior to treatment.

**Conclusion:**

There is a lack of studies which directly address COVID-19 testing of asymptomatic patients before treatment. Our systematic review showed that most of the published data favours routine test for SARS-CoV-2 before initiating systemic treatment but failed to identify a good level of evidence to support these recommendations. Based upon this review, we proposed local recommendations at our centre. Each institution should consider the pros and cons of testing asymptomatic patients, evaluating accessibility to testing resources and local epidemiology.

## Introduction

In December 2019, a rising number of patients with pneumonia of unknown cause was detected in China. A novel coronavirus named severe acute respiratory syndrome coronavirus 2 (SARS-CoV-2) was discovered to be the cause of this new type of respiratory illness, called coronavirus disease-2019, or COVID-19 [1, 2]. The rapid global dissemination of SARS-CoV-2 prompted the World Health Organization (WHO) to declare the outbreak of COVID-19 a pandemic on 11th March 2020 [3]. As of 25th June 2020, there have been approximately 9.2 million confirmed cases and more than 479,000 deaths worldwide [4]. In Brazil, there have been almost 1,220,000 confirmed cases and more than 54,000 deaths have reported [5].

Special concern is raised for patients with cancer, potentially exposed to a higher incidence of COVID-19. Moreover, they seem to have a higher risk of severe events, such as admission in intensive care units, invasive ventilation or death [6–9], and even a higher mortality rate than general population [10]. Previous oncologic treatments may also result in a higher risk of severe events [11]. These negative outcomes could potentially be explained by advanced age, immunosuppression, treatment-related effects and complications, the need for frequent hospital visits and admissions, and impaired functional status [12].

Nevertheless, a large proportion of cancer patients require prompt initiation of treatment because of potential risk of deteriorating their clinical status and presenting complications attributable to disease progression. Moreover, treatment delays may incur in a detrimental impact on the risk of recurrence, potentially compromising survival outcomes [13, 14]. Considering the fact that initiating appropriate oncological treatments for cancer patients could result in a higher risk for COVID-19-related severe events, a question about the need to test asymptomatic patients for SARS-CoV-2 infection before initiating systemic treatment has notably been raised. This systematic review will address the current evidence to answer this question.

## Methods

This systematic review was conducted based on the Preferred Reporting Items for Systematic Reviews and Meta-Analyses (PRISMA) framework [15]. The electronic databases of Pubmed, Embase, Web of Science and Cochrane Central Register of Controlled Trials (CENTRAL) were systematically searched. The included search terms were as follows: ‘COVID-19’, ‘COVID-2019’, ‘severe acute respiratory syndrome coronavirus 2’, ‘2019-ncov’, ‘coronavirus’, ‘neoplasms’, ‘cancer’, ‘drug therapy’, ‘chemotherapy’, ‘immunotherapy’, ‘manag*’. The search strategies are presented in the Appendix (Table A1). In order to optimise and expand the search results, we also examined guidelines from some of the main international institutions involved with oncology and COVID-19 research: ASCO (American Society of Clinical Oncology), ESMO (European Society for Medical Oncology), NCCN (National Comprehensive Cancer Network) and IDSA (Infectious Diseases Society of America).

We have included studies which mentioned testing strategies for SARS-CoV-2 of asymptomatic patients before receiving some systemic treatment, such as chemotherapy, targeted-therapy or immunotherapy. Only studies published in English, from 1 December 2019 to 27 May 2020 were considered eligible. We excluded studies published as case reports or systematic reviews, duplicated articles and studies focused on only surgical or paediatric patients.

Titles and abstracts identified by the search strategy were imported into Rayyan QCRI web application and blindly and independently reviewed by two investigators (G.H. and F.F.A.). Full texts of screened papers were retrieved and reviewed by the same authors for inclusion criteria. Disagreements between the two reviewers were resolved by consultation with a third investigator (G.C.J). Study design, date of publication, authors, tumour subtypes, recommendations in relation to SARS-CoV-2 test were extracted from included articles by two reviewers (G.H. and F.F.A.) using a data extraction form. Quality assessment was performed based on Oxford Centre for Evidence-Based Medicine 2011 Levels of Evidence [16]. As most of the included studies were expert consensus, meta-analysis was not performed.

## Results

### Study searching and included studies

**Figure 1.**
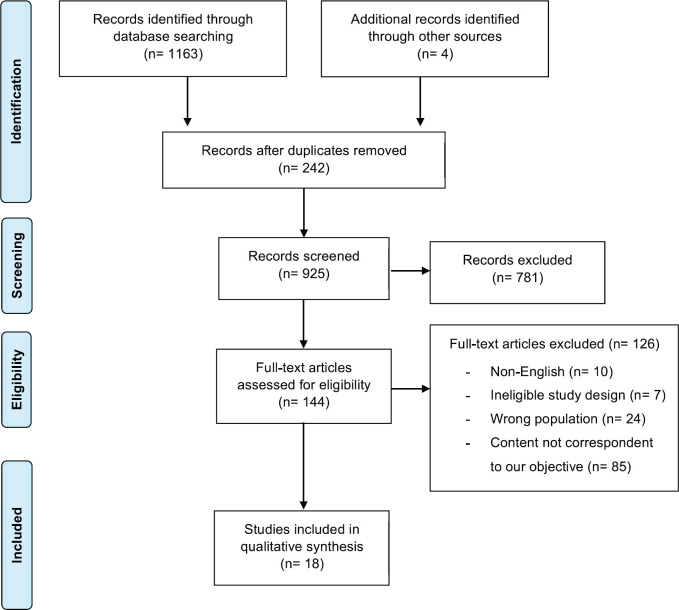
Prisma flow diagram of the article selecting process.

We identified 1,163 studies based on database searches and 4 guidelines through the literature search. After removing 242 duplicated studies and reviewing the titles and abstracts, 144 were selected for full-text evaluation. One hundred twenty-six studies were excluded, mainly due to the fact that they did not mention any strategy for testing asymptomatic cancer patients and did not fulfil the inclusion criteria. Therefore, a total of 18 articles were considered eligible and were included in the final analysis, of which 14 studies were identified based on database searches, and four were guidelines (Figure 1). Among those identified on database searches, two articles were cohort studies (level 3 of evidence) and the remaining were expert consensus (level 5).

All included studies were published in 2020. Out of 18 articles, five alluded exclusively to solid tumours, seven to hematologic malignancies and six to both groups of diseases. Two cohorts were identified. All other studies were expert consensuses.

### Cohort studies

Two cohort studies which discuss some strategies of testing for SARS-Cov-2 asymptomatic patients were identified [17, 18]. The first one was conducted in Lombardy, Italy. In order to reduce the COVID-19 infection rate, a proactive management was developed, including, among others, text messages to patients asking for symptoms, creation of a COVID-19 surveillance zone and a triage process. These procedures included not only asymptomatic patients before immunosuppressive treatments, but also surgical and hospitalised patients.

For patients considered at risk of COVID-19 infection, a triage process was started, which included a low-dose computed tomography (CT) scan of lungs. A nasopharyngeal swab real-time polymerase chain reaction (RT-PCR) test was performed on all radiologically or clinically suspected patients. For specifically asymptomatic patients who needed treatment which induced neutropenia or lymphopenia, a simplified triage process has been developed, including a swab test before the treatment. Only a preliminary result of the effects of this process was reported and did not contain the asymptomatic population outcomes separately [17].

The second cohort is from Alzahra Hospital Dubai, in the United Arab Emirates. Eighty-five asymptomatic cancer patients were enrolled. All patients underwent a RT-PCR for SARS-CoV-2 and, if positive, a chest X-ray was performed. RT-PCR was repeated until two consecutive negative results.

RT-PCR was found to be positive in 7 (8.24%) out of the 85 patients, five of them (71.4%) on systemic therapy and two who had not yet initiated therapy. Cancer types among the positive patients were breast (2 (28.6%)), colorectal (2 (28.6%)), lymphoma (2 (28.6%)), and lung (1(14.1%)). All RT-PCR positive patients eventually became symptomatic: five with mild symptoms and two with severe, requiring intensive care unit (ICU) admission. The patients with mild symptoms resumed therapy after virologic clearance (median 18 days). One of the patients admitted on ICU had lung cancer in treatment with anti-PD-L1 therapy and died of acute respiratory distress syndrome, and the other had colorectal cancer and remained hospitalised. Importantly, no patient recalled having anosmia or dysgeusia and no health care workers of the screening site were diagnosed with COVID-19. This cohort had some limitations, such as: single-centre experience, with no control group and small sample size [18].

### Expert consensuses

#### Solid tumours

Five articles discussed systematic testing for SARS-CoV-2 in asymptomatic patients with solid tumours [19–23]. Two were related to lung cancer, one to breast cancer, one to head and neck cancer and one to gynaecologic cancer. Four out of five studies considered offering the test to asymptomatic patients before starting the treatment. Table 1 summarises the recommendations of each study.

Considering that lung cancer patients can present similar clinical symptoms to COVID-19, and oncologic radiological findings can resemble or overlap with virus infections, these concordances can pose a challenge to diagnose SARS-CoV-2 infection in this specific population. Banna *et al* [19] recommend that lung cancer patients undergo testing for COVID-19 before treatment initiation and in case of a positive case of a family member or caregiver. Calabrò* et al* [20] suggest that lung cancer patients should perform SARS-CoV-2 testing before the beginning of treatment. They also emphasise that a greater availability of tests is crucial to the feasibility of this strategy.

Head and neck cancers also deserve particular considerations, mainly because of the unique location in the aerodigestive tract, a recognised region that harbours coronavirus. Chaves* et al* [21] recommend, combined with a detailed medical history and physical examination, SARS-CoV-2 testing before curative treatment, and before any hospitalisation, with the possibility of a 2-week treatment delay in case of a positive test, to protect not only the patient, but also other patients and healthcare workers.

Regarding breast and gynaecological cancer, both studies suggest, if tests are available, testing for COVID-19 before initiating immunosuppressive therapies, even for asymptomatic patients [22, 23]. Pothuri* et al* [23] also recommend that patients receiving immunotherapy should involve careful attention and have COVID-19 testing in case of respiratory symptoms, fever or new CT findings.

#### Haematologic malignancies

Patients with haematologic malignancies may have a higher risk of developing COVID-19 for multiple and potentially overlapping reasons. They often receive highly immunosuppressive treatments in addition to the cancer-related immunosuppression and are at high risk of opportunistic infections. Seven articles discuss the management of SARS-CoV-2 testing all patients before starting treatment [24–29] (Table 2). Nevertheless, each hematologic neoplasm might be associated with an intrinsic risk of COVID-19, due to diverse disease biology and related treatments. Thus, recommendations are frequently published by neoplasm subtypes separately.

At the Seattle Cancer Care Alliance, the haematologic cancer patients are not routinely being tested for SARS-CoV-2 before beginning standard chemotherapy. The capability of performing tests should be considered, and the risks of delaying effective therapies when a test is positive are uncertain. Therefore, the tests are prioritised to symptomatic patients and prior to surgeries, stem-cell transplantation (SCT), and chimeric antigen receptor (CAR) T-cell therapy In some cases, depending on the risk and the disease, such as asymptomatic patients with indolent lymphoma, can be considered deferring therapy for few weeks or months [26].

Paul *et al* [25] recommended nasopharyngeal swab and COVID-19 serology, if available, for patients with chronic lymphocytic leukaemia (CLL) who will receive myelosuppressive chemoimmuno treatments, such as fludarabine, cyclophosphamide, and rituximab, or bendamustine plus rituximab. There is a a recommendation to prefer oral regimens to minimise hospital visits (such as ibrutinib, acalabrutinib, and venetoclax).

Koffman* et al* [24] reported a survey performed by CLL specialists. Considering an unlimited capacity of COVID-19 testing, 23% suggested testing for all patients. Nevertheless, in a scenario with limited test availability, most specialists did not recommend universal testing and 62% suggested testing only symptomatic patients. In this setting, as discussed by Perini GF and cols., the testing strategy before treatment should be individualised. They also recommend not delaying treatment in aggressive lymphomas such as Burkitt lymphoma, plasmablastic lymphoma and to prioritise outpatient salvage regimens in the relapsed setting. Besides SCT should not be delayed, SARS-CoV-2 testing should also be performed both before autologous and allogenic SCT [25]. For all lymphoid malignancies, it is recommended to postpone medical appointments for patients in complete remission or for patients in which no immediate change therapy is expected. Virtual consultation/counselling is encouraged [27].

Patients with multiple myeloma (MM) may be at a higher risk of SARS-CoV-2 infection for several reasons, including disease, treatment and patient characteristics. These patients have a higher age at diagnosis and are more likely to have comorbidities. In addition, the disease can cause immunodeficiency and the treatment can aggravate the immunosuppression. three articles mentioned a strategy of COVID-19 testing these patients before treatment initiation. All three articles recommend performing SARS-CoV-2 testing for all patients before autologous SCT [28–30]. Al Saleh* et al* [28] and Terpos *et al* [30] also consider the testing before starting any immunosuppressive treatments.

#### Guideline recommendations

Four guidelines of the main international institutions involved with oncology and COVID-19 research were selected and discuss systematic testing for SARS-CoV-2 in asymptomatic cancer patients (Table 3).

All ASCO recommendations are based upon available testing resources and laboratory capacity; therefore, they allocated cancer patients into priority groups to be tested. Asymptomatic individuals prior to receipt of immunosuppressive therapy are priority 3 for testing, after symptomatic and hospitalised patients. ASCO recommends that all new patients prior to receiving cytotoxic chemotherapy, SCT, biologic therapy, immunotherapy or high-dose corticosteroids, should be tested 72 to 48 hours before commencing treatment and, during treatment, every patient should be screened with a standardised questionnaire. If possible, they recommend the test occurs at a site other than the hospital or cancer care clinic [31].

ESMO consensus also suggests that SARS-CoV-2 RT-PCR testing should be recommended to all patients undergoing chemotherapy or immunotherapy and ideally before each treatment cycle. Considering the availability of resources in each cancer centre, and limited access to testing, RT-PCR should be offered only to symptomatic patients [32].

IDSA recommends routinely testing all cancer patients, including asymptomatic individuals, before immunosuppressive therapies, regardless of a known exposure to COVID-19. IDSA also advises that testing should ideally be performed as close to the planned treatment as possible (within 48–72 hours). In the outpatient setting, where patients require frequent visits to the clinic or infusion room, IDSA recommends regular screening with a standardised questionnaire for symptoms and known exposures between visits [33].

Opposed to the other guidelines, NCCN does not recommend testing for all asymptomatic patients being considered for systemic therapy. Instead, they suggest several measures that can be implemented to provide cancer care safely, such as considering alternative dosing schedules, switching to oral therapies (if available) and administration at home through home care nurses. There is only one exception, namely, patients with hematologic malignancies and/or undergoing SCT [34].

## Discussion

Cancer centres all over the world are dealing with different challenges regarding cancer care during the COVID-19 pandemic, which go from lower availability of diagnostic/workup studies to deciding which patients should go in-person to the centre and even if treatments should be postponed [13, 35]. In a large cohort including only oncologic patients, approximately 4% tested positive for SARS-CoV-2 and were reported as asymptomatic [10]. The fact that cancer patients represent a population at a higher risk of developing COVID-19 makes the decisions regarding the patients more critical. A mortality rate of 13% among cancer patients has been described [10], compared to 1.4% in an unselected patient population [2]. In patients with confirmed SARS-CoV-2 infection, delaying systemic therapies should be strongly considered [31, 36]. Therefore, deciding whether to test asymptomatic patients before starting systemic treatment is one of the oncologists’ daily practice issues.

Our systematic review failed to identify a good level of evidence to support the routine test for SARS-CoV-2 before initiating systemic treatment even though this routine was uniformly recommended among the selected publications. This brings some limitations that should be considered when interpreting the results of this review.

A positive SARS-CoV-2 RT-PCR test could guide the decision of delaying the systemic oncologic treatment and possibly avoiding COVID-19 severe complications in an immunosuppressed patient [12], but this assumption has not been confirmed to date. In general population, COVID-19 asymptomatic patients seem to have less decrease in CD^4+^ lymphocyte counts and a shorter duration of viral shedding, but this has not yet been reported in cancer patients [37]. In addition, confirmed SARS-CoV-2 infected patients must be isolated, protecting other immunocompromised patients who frequent the cancer care facilities and the health workers against COVID-19 [38]. Testing patients before initiating a systemic treatment could also prevent legal issues in case of poor outcomes due to COVID-19 during chemotherapy nadir, for instance.

In contrast, there are negative points in performing the SARS-CoV-2 test before starting the systemic treatment. The RNA test for prevalent infection is an essential part of the diagnosis strategy [39]. The RT-PCR method for viral detection is usually characterised by fast results, high sensitivity and specificity. The clinical sensitivity of RT-PCR tests for the new coronavirus varies from 50% to 80% [40–42]. However, it is worth mentioning that this is true when we are dealing with symptomatic patients, but the role of testing for asymptomatic cancer patients remains unclear [43]. In a prospective study with 400 asymptomatic health-care workers weekly tested for SARS-CoV2, only 12 patients who tested positive had no symptoms the week before or after the test and were diagnosed with COVID-19 [44]. FDA granted accelerated emergency use authorisation for COVID-19 RT-PCR test for patients with suspected upper respiratory tract infection, but asymptomatic patients were exclusion criteria for testing [45].

A review analysed seven previously published studies providing results on 1,330 RT-PCR testing for SARS-CoV-2 and time since onset of symptoms or exposure. The results demonstrated over the 4 days of infection before the typical time of symptom onset, the probability of a false-negative result in an infected person decreased from 100 on day 1 to 67% (95% CI, 27%–94%) on day 4. On the day of symptom onset, the median false-negative rate was 38% (95% CI, 18%–65%). Thus, the false-negative rate for SARS-CoV-2 RT-PCR testing is highly variable especially within the days of no symptoms [46].

Overall, potential RT-PCR vulnerabilities are related to multiple pre-analytical and analytical factors [47]. The main points highlighted were the lack of standardisation for the collection of material, insufficient viral load, and specimens, time and conservation of samples, use of non-validated tests, contamination during the procedure, disease incubation period and risk of recombination and active mutation [48].

Health systems can be overwhelmed during a viral pandemic emergency in all areas, from preventive and diagnostic measures to resources for treatment [49]. The rapidly growing imbalance between supply and demand for medical resources in many countries represents a worrying issue [50]. Hence, in countries like Brazil, where financial resources directed to health care are scarce, the expense of recommending testing for everyone, including asymptomatic individuals, should be taken into account.

Other concerns on this theme would be the ideal frequency for performing RT-PCR and the possibility of patients being infected between the test day and the medical appointment. In this systematic review, we were unable to answer this question, and hence, these are matters that each service tended to follow its own recommendation.

As mentioned, the main oncological societies in the world have developed guidelines to mitigate the negative effects of the COVID-19 pandemic on the diagnosis and treatment of cancer patients. The scope of these proposed guidelines is to categorise patients as high, medium or low priority, based on the clinical criteria intrinsic to the patient and the disease, in order to plan their management course accordingly [31–34]. That said, a question is raised when we come across a positive test an asymptomatic cancer patient. In our view, it is necessary to make an individual risk-benefit assessment, evaluating case-by-case, and proceed with cancer treatment whether the benefits outweigh the risks. Table 4 summarises the pros and cons of RT-PCR test for asymptomatic cancer patients.

Given the current unusual situation, pragmatic approaches are required to deal with the challenges of treating cancer patients, without risking their care. Clear communication and education about hand hygiene, face masks, infection control measures, signs and symptoms of the COVID-19, high-risk travel or exposure and the importance of reporting new symptoms to their health care providers should be reinforced.

Due to the paucity of structured data to routinely submit patients initiating treatment to COVID testing and the high false-negative rates, our institution has considered among our group of oncologists and haematologists all the pros and cons in regard to SARS-CoV-2 testing for asymptomatic patients before initiating oncologic treatment, and decided to follow a selective approach. We do not recommend routinely testing all cancer patients before initiating systemic treatments. Based upon the highest frequency of severe events, we advise testing asymptomatic patients prior to SCTs, any systemic treatment of hematopoietic neoplasms, concurrent chemoradiation regimens (presenting with high frequency of post-treatment lymphopenia) or in those patients with lung cancer or head and neck cancer initiating systemic treatments with immunosuppressive properties.

**Figure A1.**
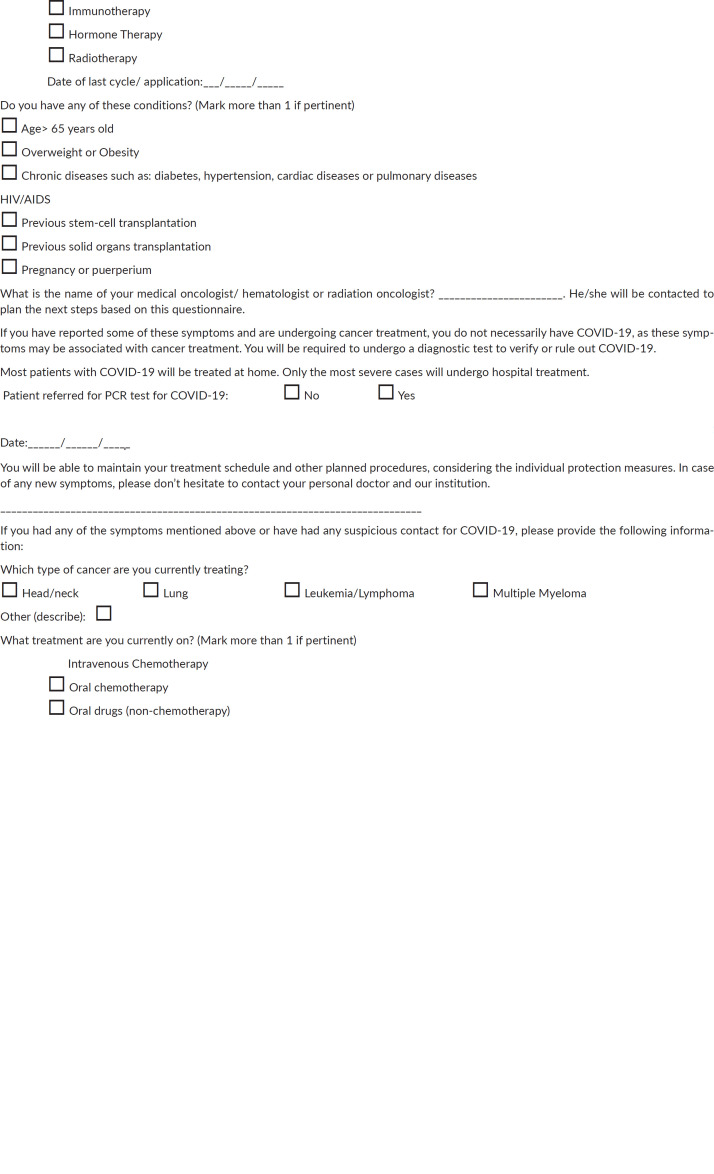
Questionnaire.

Suggested steps to reduce the risk of exposure include a call to the patient 48 hours prior to their scheduled appointment and on the day of treatment to screen for any symptoms associated with COVID-19, contact history, as well as recent travel history. During treatment, all patients should be screened 48 hours before each new cycle of therapy. We strongly recommend a detailed questionnaire that embraces all relevant aspects of COVID-19. The questionnaire which we proposed in our institution is presented in the Appendix Figure A1. Based on screening results, health care workers should test any patient identified as having whichever suspected symptom, besides considering including a chest computed tomography scan. The combination of clinical features with RT-PCR might increase the pretest sensitivity. As testing becomes more widely available, it may be reasonable expand this approach to test all asymptomatic patients who will be receiving immunosuppressive anticancer therapy.

## Conclusion

This systematic review has demonstrated that there is a lack of robust evidence to support SARS-CoV-2 testing of asymptomatic patients before cancer treatment even though this routine is mostly suggested among the guidelines and expert consensuses.

The crucial point is to individualise the centres and their socioeconomic circumstances, considering the accessibility to testing resources, laboratory capacity and regional epidemiology. Based upon this review our group proposed our own recommendations. We plan to collect and publish prospective data after adopting our institution`s consensus. Finally, it is important to mention that these recommendations may change in the future, depending on new scientific evidence.

## Funding

This research did not receive any specific grant from funding agencies in the public, commercial, or not-for-profit sectors.

## Conflicts of interest

The authors declare that they have no conflicts of interest.

## Figures and Tables

**Table 1.** Recommendations of solid tumour studies.

Author	Date of publication	Cancer site	Recommendation
Banna *et al* [19]	15 April 2020	Lung	SARS-CoV-2 testing before the beginning of treatment, in case of a positive case of a family member or caregiver
Calabrò *et al* [20]	9 April 2020	Lung	SARS-CoV-2 testing before the beginning of treatment
Chaves *et al* [21]	24 April 2020	Head and neck	SARS-CoV-2 testing before curative treatment
Curigliano *et al* [22]	16 April 2020	Breast	SARS-CoV-2 testing before initiating immunosuppressive therapies
Pothuri *et al* [23]	23 April 2020	Gynaecological	SARS-CoV-2 testing prior to cancer directed therapy

Abbreviations: SARS-CoV-2 = Severe acute respiratory syndrome coronavirus 2.

**Table 2.** Recommendations of hematologic malignancies studies.

Author	Date of publication	Hematologic neoplasm	Recommendation
Percival* et al* [26]	5 May 2020	Not specified	SARS-CoV-2 testing are not routinely performed before standard chemotherapy Tests should be considered prior to SCT and CAR T-cell therapy
Paul* et al* [25]	11 May 2020	Leukaemia	SARS-CoV-2 testing for patients with CML on TKI or with CLL who will receive myelosuppressive chemoimmuno treatment, such as FCR or bendamustine plus rituximab; SARS-CoV-2 testing before allogenic SCT
Koffman* et al* [24]	30 April 2020	CLL	Survey with CLL specialist: considering an unlimited capacity of COVID-19 testing, 23% suggested for all patients; in a scenario with limited test availability, most specialists did not recommend universal testing and 62% suggested the test only to symptomatic patients
Perini* et al* [27]	17 April 2020	Lymphoid malignancies	Testing before treatment is unclear and testing strategy should be individualised
Al Saleh* et al* [28]	17 April 2020	MM	SARS-CoV-2 testing before starting treatment and autologous SCT
Malard* et al* [29]	27 April 2020	MM	SARS-CoV-2 testing before autologous SCT
Terpos* et al* [30]	13 May 2020	MM	SARS-CoV-2 testing before starting a new treatment line, or autologous SCT

Abbreviations: CAR = chimeric antigen receptor; CML = chronic myeloid leukaemia; CLL = chronic lymphocytic leukaemia;

FCR = fludarabine, cyclophosphamide, and rituximab; MM = multiple myeloma; SCT = stem-cell transplantation.

**Table 3.** Guideline recommendations.

Guideline	Recommendation
ASCO [31]	After considering availability of tests and laboratory capacity: asymptomatic new patients should be tested for SARS-CoV-2 48-72h prior to initiating treatment with cytotoxic chemotherapy, stem cell transplantation, long acting biologic therapy, cellular immunotherapy or high-dose corticosteroids; screening 48-72h prior to each new cycle of treatment with a standardised questionnaire
ESMO [32]	SARS-CoV-2 RT-PCR testing should be proposed to all patients undergoing chemotherapy or immunotherapy and ideally before each treatment cycle; If availability of tests is limited, RT-PCR should be offered only for symptomatic patients; serology should be offered to all cancer patients. If not available, limit tests to all patients undergoing chemotherapy or immunotherapy or any other active anti-cancer treatment
IDSA [33]	SARS-CoV-2 testing asymptomatic patients before immunosuppressive procedures (i.e. cytotoxic chemotherapy, solid organ or stem cell transplantation, long acting biologic therapy, cellular immunotherapy or high-dose corticosteroids) regardless of known exposure to COVID-19, 48-72h prior to treatment initiation; screening with a standardised questionnaire for symptoms and exposure should be performed in between hospital visits.
NCCN [34]	For hematologic malignancies and stem cell transplant: testing for SARS-CoV-2 should be considered asymptomatic patients before receiving chemotherapy that will result in significant and prolonged immunosuppression.

Abbreviations: ASCO = American Society of Clinical Oncology; ESMO = European Society for Medical Oncology; IDSA = Infectious Diseases Society of America; NCCN = National Comprehensive Cancer Network.

**Table 4.** Pros and cons of RT-PCR test for asymptomatic cancer patients.

SARS-CoV-2 RT-PCR test for asymptomatic cancer patients	
Pros	Cons
To avoid (potential) COVID-19 severe complications in cancer patient*	Value in asymptomatic patients not established
To protect other immunocompromised patients	Increased number of false-negative tests
To protect healthcare workers	To encumber health systems with expenditure of medical resources
To decrease disease dissemination	Not standard approach after a positive test
To avoid legal issues	No ideal frequency for performing RT-PCR

* Not demonstrated based on prospective data.

## Appendix

**Table A1.** Search strategies.

Database	Search terms
Pubmed	(‘COVID-19’ [All Fields] OR ‘COVID-2019’ [All Fields] OR ‘severe acute respiratory syndrome coronavirus 2’ [Supplementary Concept] OR ‘severe acute respiratory syndrome coronavirus 2’ [All Fields] OR ‘2019-nCoV’ [All Fields] OR ‘SARS-CoV-2’ [All Fields] OR ‘2019nCoV’ [All Fields] OR (‘coronavirus’ [MeSH Terms] OR ‘coronavirus’[All Fields])) AND (‘neoplasms’ [MeSH Terms] OR ‘neoplasms’ [All Fields] OR ‘cancer’ [All Fields])) AND (‘drug therapy’ [Subheading] OR (‘drug’ [All Fields] AND ‘therapy’ [All Fields]) OR ‘drug therapy’ [All Fields] OR ‘chemotherapy’ [All Fields] OR ‘drug therapy’ [MeSH Terms] OR (‘drug’ [All Fields] AND ‘therapy’ [All Fields]) OR ‘chemotherapy’ [All Fields] OR (‘immunotherapy’ [MeSH Terms] OR ‘immunotherapy’ [All Fields])OR MANAG*)
Embase	(‘COVID 19’/exp OR ‘COVID 19’ OR ‘severe acute respiratory syndrome coronavirus 2’/exp OR ‘severe acute respiratory syndrome coronavirus 2’ OR ‘COVID 2019’ OR ‘2019 ncov’ OR ‘sars cov 2’ OR 2019ncov OR ‘coronavirus’/exp OR coronavirus) AND ((‘neoplasm’/exp OR neoplasm) OR (‘cancer’/exp OR cancer)) AND ((‘drug’/exp OR drug) AND (‘therapy’/exp OR therapy) OR ‘chemotherapy’/exp OR chemotherapy OR ‘immunotherapy’/exp OR immunotherapy OR manag*)
Web of Science	ALL = (COVID-19 OR COVID-2019 OR ‘severe acute respiratory syndrome coronavirus 2’ OR 2019-ncov OR coronavirus ) AND ALL = (neoplasms OR cancer) AND ALL= (‘drug therapy’ OR chemotherapy OR immunotherapy OR manag*)
Central	(COVID-19 in All Text OR COVID-2019 in All Text OR ‘severe acute respiratory syndrome coronavirus 2’ in All Text OR coronavirus in All Text) AND (neoplasms in All Text OR cancer in All Text) AND (‘drug therapy’ in All Text OR chemotherapy in All Text OR immunotherapy in All Text OR MANAG$ in All Text)

Abbreviation: Central = Cochrane Central Register of Controlled Trials.
